# Supplementary material for: The cost-effectiveness of a program to reduce intrapartum and neonatal mortality in a referral hospital in Ghana
Source: PLoS One. 2020 Nov 13;15(11):e0242170. doi: 10.1371/journal.pone.0242170 (PMC7665827; doi:10.1371/journal.pone.0242170)
Supplement: Attachment — Submitted filename: Response to Reviewers.docx [file pone.0242170.s001.docx]

**Response to Reviewer Comments**

| **Reviewer Comment** | **Response to Reviewer** | **Page Number(s)** |
| --- | --- | --- |
| **Academic Editor Review** | | |
| Please ensure that your manuscript meets PLOS ONE's style requirements, including those for file naming | Figure file names and author affiliations have been updated in accordance with PLOS ONE’s style requirements. | 1 |
| In your ethics statement in the manuscript and in the online submission form, please clarify whether informed consent was actually waived by the ethics board as part of your IRB approval | Informed consent was waived by the ethics board as part of the study’s IRB review. We have clarified this in the online submission form and within the manuscript. | 7 |
| We noticed you have some minor occurrence of overlapping text with the following previous publication(s), which needs to be addressed .In your revision please quote or rephrase any duplicated text outside the methods section. | The manuscript under review was carefully checked by hand and using two separate online platforms, against the previous publication. All instances of overlapping text have been addressed. | Throughout manuscript |
| We note that you have included the phrase “data not shown” in your manuscript. Unfortunately, this does not meet our data sharing requirements…Please add a citation to support this phrase or upload the data that corresponds with these findings to a stable repository (such as Figshare or Dryad) and provide and URLs, DOIs, or accession numbers that may be used to access these data. | We have added the appropriate data points and citation to the manuscript. | 22 |
| PLOS requires an ORCID iD for the corresponding author in Editorial Manager on papers submitted after December 6th, 2016. Please ensure that you have an ORCID iD and that it is validated in Editorial Manager. | The corresponding author has uploaded his ORCID iD in the Editorial Manager (<https://orcid.org/0000-0003-3410-4441>). |  |
| Please amend either the abstract on the online submission form (via Edit Submission) or the abstract in the manuscript so that they are identical | We will address this discrepancy when re-uploading. |  |
| Please include captions for your Supporting Information files at the end of your manuscript, and update any in-text citations to match accordingly. Please see our Supporting Information guidelines for more information:[http://journals.plos.org/plosone/s/supporting-information](about:blank). | We do not have any Supportive Information files. All related data has been uploaded to figshare, and the associated access information is included in the submission  Private link for reviewers: [https://figshare.com/s/b095e531f0e169fa67fd](https://figshare.com/s/b095e531f0e169fa67fd)  DOI: 10.6084/m9.figshare.13102913 |  |
| **Reviewer #1** | | |
| The term “systems strengthening” is an over-claim and should be removed from the title and throughout. This is not really what I would call a “systems strengthening” intervention. Judging by table 1 it was a training intervention largely or solely delivered by foreign experts. It only involved one tertiary referral hospital (not the health system as a whole), and involved 27 health professionals travelling to Ghana from the USA / UK and working for 484 days at GARH during the project. This is a rather top-down approach. No mention is made of training local staff to deliver the training to their colleagues to make this sustainable, or of making the training more widely available throughout the health system. Mention is made of a few other hospitals, but no mention is made of primary care or other parts of the health system. Why was a “train the trainers” approach not taken, which would have made the training sessions much cheaper, more sustainable, and more cost-effective? $1100 per day is probably several orders of magnitude greater than doctors in Ghana are paid – as such there is great scope for making this much more cost-effective by training trainers locally and reducing the massive foreign input, which as the authors mention is not scalable or replicable throughout Africa. | The term “systems strengthening” has been removed from the title. However, the authors feel that the content constitutes a hospital-based systems strengthening intervention to improve newborn health. This language has been added throughout. A shift in the funder’s priorities allowed only basic resuscitation and newborn care training to be conducted in the four other regional hospitals, therefore, the hospital-based systems strengthening component could only be implemented at GARH. This piece, however, went beyond a training intervention and included the selection of local champions who had the capacity to deliver trainings, leadership development, and continuous quality improvement. A more detailed description of the intervention is provided elsewhere, as cited in this manuscript (Ramaswamy R, Iracane S, Srofenyoh E, Bryce F, Floyd L, Kallam B, et al. Transforming maternal and neonatal outcomes in tertiary hospitals in Ghana: An integrated approach for systems change. J Obstet Gynaecol Canada [Internet]. 2015;37(10):905–14. Available from: http://dx.doi.org/10.1016/S1701-2163(16)30029-9).  Published examples with regards to the obstetric triage and leadership interventions are demonstrated at Goodman DM, Srofenyoh EK, Ramaswamy R, Bryce F, Floyd L, Olufolabi A, et al. Addressing the third delay: Implementing a novel obstetric triage system in Ghana. BMJ Glob Heal [Internet]. 2018 [cited 2019 Jun 21];3. Available from: http://gh.bmj.com/ and Pfeiffer E, Owen M, Pettitt-Schieber C, Van Zeijl R, Srofenyoh E, Olufolabi A, et al. Building health system capacity to improve maternal and newborn care: A pilot leadership program for frontline staff at a tertiary hospital in Ghana. BMC Med Educ [Internet]. 2019 Dec 11 [cited 2020 Feb 25];19(1):52. Available from: https://bmcmededuc.biomedcentral.com/articles/10.1186/s12909-019-1463-8. | 1 |
| The second major point is that little information is provided on other ongoing interventions or changes which could also have had an impact on neonatal mortality. The authors state “there could be demand-side and supply-side factors impacting these data.” – are they aware of any other specific interventions or factors taking place in their study site in 2012-2016 which could have impacted on neonatal or perinatal mortality? Were there changes in health insurance payments for example? The discussion states “partnership with a local bank led to an investment to renovate the NICU” – was this taken into account in the costs? This could also explain part of the reduction in mortality | During the time of intervention implementation at GARH, select providers may have attended opportunistic trainings on various topics, and there was a separate research team intermittently on-site collecting data for a pre-eclampsia study. However, no additional activities were taking place at GARH during the intervention period that were directly related to improving neonatal care throughout the system. We have added this clarification in the manuscript.  We thank the reviewer for pointing out the costs associated with the NICU renovation. We discovered we had omitted this expense that amounted to $4980.28. This amounts to 0.3% of the total costs of the project, and this omission will not affect our findings or conclusions. In consultation with the editor, we have mentioned this as a footnote on page 10. | 22  10 |
| The discussion states “It is likely, therefore, that the “no intervention” counterfactual could have had increasing mortality rates as a consequence of increasing prevalence of high-risk cases” – but is there any data to support this, on prevalence of high-risk cases in each year? If so this data should be presented. Certainly overall numbers were falling so this would not support this argument. The total number of babies delivered reduced from 11338 in 2012 to 8129 in 2016 (a 28% reduction). Why is this? Could this also explain the reduction in mortality, as the hospital would have been less stretched and there would have been more resources available per baby (assuming human resources and other factors remained constant)? | The reviewer brings up a good point regarding the falling delivery numbers. In 2013, there was both a doctor’s strike and the closing of the maternity unit due to construction that accounted for the decrease in admissions. Other than that year, the numbers of newborn admissions to the NICU at GARH was relatively uniform: 2012 (1410), 2013 (1102), 2014 (1305), 2015 (1394) and 2016 (1267). In addition, the prevalence of high-risk maternal conditions (pre-eclampsia and obstetric hemorrhage) were relatively consistent - 2012 (23.5%), 2013 (21.6%), 2014 (21.5%), 2015 (20.7%) and 2016 (25.7%). Incidentally, in 2011 there were 9599 babies delivered, 940 NICU admissions and the NMR was 38.04. It is unclear why births increased to such an extent in 2012, but the NMR in 2011 was even higher (38.04) than in 2012 (31.12), the baseline year. | 23 |
| The authors claim this was a “quality improvement” intervention but very little information is provided (apart from table 1) of what was done to improve quality, and whether anything other than mortality was measured to see whether quality did indeed improve after the numerous training sessions. For example were any audits done of neonatal resuscitation to see whether this was being better implemented after the training? What key performance gaps were identified? Were these measured again after the training to see if they had improved? Did clinicians perform maternal and perinatal death surveillance and reviews? If so which avoidable factors were identified, what recommendations were made to address them, and were these recommendations implemented? | A detailed description of the systems strengthening component of MEBCI that was implemented at GARH is provided in a previous manuscript, including the quality improvement intervention (Ramaswamy R, Iracane S, Srofenyoh E, Bryce F, Floyd L, Kallam B, et al. Transforming maternal and neonatal outcomes in tertiary hospitals in Ghana: An integrated approach for systems change. J Obstet Gynaecol Canada [Internet]. 2015;37(10):905–14. Available from: http://dx.doi.org/10.1016/S1701-2163(16)30029-9). This paper is cited throughout the present manuscript, particularly in the description of program activities on page 6, and provides an in-depth overview of the components of MEBCI and how they were measured.  Other measures of quality, including timeliness of care, associated with this intervention have been published (Goodman DM, Srofenyoh EK, Ramaswamy R, Bryce F, Floyd L, Olufolabi A, et al. Addressing the third delay: Implementing a novel obstetric triage system in Ghana. BMJ Glob Heal [Internet]. 2018 [cited 2019 Jun 21];3. Available from: http://gh.bmj.com/; Floyd L, Bryce F, Ramaswamy R, Olufolabi A, Srofenyoh E, Goodman D, et al. The introduction of a midwife-led obstetric triage system into a regional referral hospital in Ghana. Midwifery. 2018;61(February 2017):45–52; and Owen MD, Floyd L, Bryce F, Ramaswamy R, Pearson N, Morgan K, et al. Implementation of a triage system to reduce wait time and prioritise care for high-risk obstetric patients in a regional hospital in Accra, Ghana. Lancet Glob Heal [Internet]. 2016 Apr 1 [cited 2020 Oct 11];4:S16. Available from: https://linkinghub.elsevier.com/retrieve/pii/S2214109X16300213).  Audits of neonatal resuscitation were conducted which addressed key performance gaps and this has been recently published (Brathwaite KP, Bryce F, Moyer LB, Engmann C, Twum-Danso NAY, Kamath-Rayne BD, et al. Evaluation of two newborn resuscitation training strategies in regional hospitals in Ghana. Resusc Plus [Internet]. 2020 Mar [cited 2020 Sep 10];1–2:100001. Available from: https://www.sciencedirect.com/science/article/pii/S2666520420300011). | 6-7 |
| The cost-effectiveness calculation is impressive but parts of it are not intelligible to a non-health economist. I would recommend making this clearer to those of us who are not experts in health economics. For example, the YLL equation does not include an explanation as to what the letters stand for. | An explanation of variables in the YLL formula was included, and the variables were indicated within the text to provide additional context for the formula. | 13 |
| Figure 3 and Table 5 need further explanation to make them clearer to non-specialists. | Additional legend and footnotes have been added to Table 5 to provide explanation of what information is included. Additional language has been added to provide clarification on the purpose of Figure 3. | 18, 20 |
| Regarding cost-effectiveness, I think the data actually makes it fairly clear that the model of sending out many foreign experts to conduct training (which is often done as part of health partnerships) is probably NOT cost-effective by African standards and that more cost-effective models are needed. The cost-effectiveness threshold stated is theoretical but has it ever been used by any country in Africa? It seems to me more akin to thresholds used in high-income countries. Just one simple example: a dose of surfactant costs about $90 which can save the live of a premature baby with respiratory distress. According to the authors’ calculations this would translate to about $3 per DALY (assuming a discounted life expectancy of about 30 years). Yet this is deemed to be too expensive in most if not all African countries, and as a result surfactant is either not available at all, or maybe only in private hospitals which are not affordable to the majority. Was surfactant available at the Ghanaian hospital, and if not, how much would it have cost, and how many lives could have been saved in comparison with the reported intervention? It seems to me highly improbable that any African country would be willing to spend $139 per DALY on any intervention. The discussion should recognise this and should also consider and discuss ways in which the intervention could have been made more cost-effective and replicable. There should be a greater focus on lessons learned and which parts of this intervention might be relevant to other settings and might be scalable. | As noted by the reviewer, there are established thresholds for cost-effectiveness that are commonly used throughout high-income countries like the US and UK, as discussed in the manuscript. The authors chose to use a threshold based on GDP, as well as the league table approach by the World Bank, as these approaches are more commonly used in low- and middle-income country settings to determine cost-effectiveness. The GDP threshold for cost-effectiveness has been widely used in research across African countries, and additional text and citations have been added to the manuscript in support of this approach.  Additionally, the intervention discussed in this manuscript goes beyond foreign experts conducting training. As noted in a previous response, a detailed overview of the intervention is provided in a previous manuscript and highlights the complexity of the systems strengthening approach.  To address the reviewer’s question, surfactant was not available, however, continuous positive airway pressure (CPAP) ventilation was introduced into the NICU to address prematurity as part of this intervention and has been locally sustained. It is unreasonable and unsafe to provide equipment, techniques or pharmacologic agents alone, without appropriate on-site instruction and mentoring by knowledgeable and experienced clinicians, and in our opinion, this is why other training approaches have often failed. The foreign experts were clinicians who coached and mentored at the point of care to ensure that the training concepts translated into the safe provision of care. The time and effort spent developing higher level clinical and operational skill sets for front line staff at GARH is being locally sustained and expanded.  By strengthening the ability of GARH, at a systems level, to provide improved neonatal care, the cost per DALY ultimately results in ongoing benefits to the hospital due to increased capacity. The intervention discussed in this analysis represents initial implementation work, and learning from this study is now being scaled up in other hospitals under a model led by Ghana Health Services. Text to clarify the ongoing work in Ghana, as a result of this cost-effective model, has been added to the manuscript. | 15, 23 |
| “NMR decreased by 19.4% in 5 years and neonatal deaths reduced from 353 to 204 per 1,000 live births” - According to table 4 these are absolute numbers, not a rate per 1000 live births. The rate per 1000 live births is reported as 31.12 and 25.08. | The text has been updated. | 17 |
| Table 5: to a non health economist it is not clear what the figures mean in square brackets in the title row of the table, such as [0.3, 1]. Please explain this in the legend. | A legend was added to Table 5 to explain the different calculations for YLL represented within the table. | 19 |
| p21: “Fourth, systems strengthening interventions are generally time intensive; thus, programs such as this may be difficult to replicable” change to “Fourth, systems strengthening interventions are generally time intensive; thus, programs such as this may be difficult to replicate” | The text has been edited from “replicable” to “replicate.” | 24 |
| **Reviewer #2** | | |
| **[Introduction]** Could the authors elaborate on this sentence on page 5 “However, many QI programs focus on a limited range of problems and leave unaddressed gaps.” It would also be worth highlighting some the QI interventions aimed at reducing neonatal mortality in SSA, with regards to effectiveness and costs. See for example (Cavicchiolo, M.E, et al., 2016. Reduced neonatal mortality in a regional hospital in Mozambique linked to a Quality Improvement intervention. BMC pregnancy and childbirth, 16(1), p.366.) | We have elaborated on this by citing the opinion of QI researchers that most QI programs are time-bound and project-based, and do not build capacity for continual improvement(Dixon-Woods M, Martin GP. Does quality improvement improve quality? Futur Hosp J [Internet]. 2016 [cited 2020 Oct 14];3(3):191–4. Available from: http://sites.bu.edu/ciis/files/2016/10/Dixon-Woods.pdf). This is the point we were trying to make. While we could provide multiple examples of QI interventions in SSA, they only reinforce our assertion. What we want to emphasize is that despite the prevalence of QI interventions reported in the literature, they are all individual studies and do not represent a capacity building program such as the one described in this paper. | 5 |
| **[Introduction]** Some specifics on how does this study expand on/fill specific gaps in the neonatal mortality intervention lit? | Additional text has been added discussing the gap in the literature filled by this analysis. | 5-6 |
| **[Introduction]** Authors should include specific objectives of this economic evaluation. | The specific objective of this study was added to the Introduction. | 5 |
| **[Introduction]** In the final paragraph [of the introduction], it would be important to highlight the relevance of this CEA for health policy makers and programme planners. | After reflecting on comments from reviewers, the authors recognize that the study lends itself to policy- and decision-makers. Subsequent to the preparation of this manuscript, Ghana Health Services is implementing this particular intervention and currently working to scale the model to three other regional facilities throughout the country. The reviewer has highlighted the need to discuss the impact of this analysis for policy makers, and additional language has been added to the manuscript to reflect this key point. | 22 |
| **[Introduction]** Could the authors highlight what part of the current programme was informed by the previous programme evaluated by this group (Goodman et al., 2017)? It is unclear how different or similar the previous programme is to the current programme. A diagram/appendix highlighting what each component targeted is needed, particularly the health systems strengthening components (e.g. under QI- what aspects did this cover?) | The work previously done by Goodman et al. (2017) analyzed the cost-effectiveness of previous efforts of the Kybele-GHS partnership focused primarily on maternal and fetal outcomes. The focus of this intervention was primarily on newborns. Additional text has been added to the Introduction to provide context around the long-standing relationship between Kybele and GHS and previous projects. A detailed description of the systems strengthening component of MEBCI that was implemented at GARH is provided in a previous manuscript (Ramaswamy R, Iracane S, Srofenyoh E, Bryce F, Floyd L, Kallam B, et al. Transforming maternal and neonatal outcomes in tertiary hospitals in Ghana: An integrated approach for systems change. J Obstet Gynaecol Canada [Internet]. 2015;37(10):905–14. Available from: http://dx.doi.org/10.1016/S1701-2163(16)30029-9). This article by Ramaswamy et al. (2015) provides a figure outlining the different systems strengthening program components, including quality improvement.  The current intervention was built on key learnings from the previous intervention, but added more structure and a better data collection system. Details of the previous intervention can be found at: Srofenyoh EK, Kassebaum NJ, Goodman DM, Olufolabi AJ, Owen MD. Measuring the impact of a quality improvement collaboration to decrease maternal mortality in a Ghanaian regional hospital ☆. Int J Gynecol Obstet [Internet]. 2016 [cited 2020 Oct 4];134:181–5. Available from: http://dx.doi.org/10.1016/j.ijgo.2015.11.026 and Srofenyoh E, Ivester T, Engmann C, Olufolabi A, Bookman L, Owen M. Advancing obstetric and neonatal care in a regional hospital in Ghana via continuous quality improvement. Int J Gynecol Obstet [Internet]. 2011 [cited 2020 Oct 4];116:17–21. | 6 |
| **[Methods]** In the abstract, this study is described as “quasi-experimental and time-sequence”- could the authors elaborate on this in the methods. What about this makes it a quasi-experimental /time-sequence and not simply a pre-post evaluation? | Additional text has been added to the methods section to justify the description of a quasi-experimental, time-sequence design. | 13 |
| **[Methods]** Perspective & costs estimates- a stronger justification on why a perspective of the NGO was taken and why costs are not estimated using Ghanaian rates- particularly volunteer HCWs? Surely findings have major implications for the Ghanaian MOH. For sustainability reasons, it would be beneficial to the MOH on what are the implications for scale-up of this programme/integration this within routine services. I would recommend that the authors present primary findings taking into account Ghanaian salary rates. An appendix could be inserted for costs estimates calculated using USA HCW salary rates. | The primary implementer of this intervention was an NGO, informing the decision to present the cost-effectiveness analysis from the NGO perspective. The primary purpose for utilizing US estimates for volunteer time value was to represent a worst-case scenario for the cost-effectiveness analysis. Had the authors used Ghanaian salary estimates, the analysis would have shown the intervention as even more cost-effective than we found it to be in the present manuscript. The authors prioritized the use of actual costs associated with the implementation of the intervention, rather than inflating the findings through the use of Ghanaian salary estimates for providers’ time. Given the capacity-building nature of this intervention, ongoing and future efforts do not rely on volunteered time from international experts; the intervention can be implemented going forward by local Ghana Health Services employees, thus making it even more cost effective. | 23 |
| **[Methods]** Table 2 should be included in the results and expanded on | Table 2 outlines the reported program costs and associated inflation-adjusted costs. This information was used to inform the analysis, and we feel thus belongs in the Methods section of the paper, since it is input data rather than output data. Footnotes have been added to Table 2 to expand upon what was included within each component of the budget. | 10 |
| **[Methods]** Some elaboration is needed on which specific data sources were used to extract key outcome data. Where any of these routine data sources? | Information on the data sources for outcome data was added to the manuscript. | 14 |
| **[Methods]** The authors should consider some assessment of affordability. Part of the modeling should consider what the estimated financial cost would be if the programme was run by the MOH under routine set-up, using various scenarios. | From an affordability standpoint, the purpose of this intervention is to strengthen capacity at the hospital-level. Building capacity to engage in ongoing leadership and QI activities has long-term benefits from a cost perspective. As this work serves as the initial implementation of a model, ongoing efforts to scale this model will allow for it to be implemented locally at lower costs.  With that in mind, conducting an additional analysis related to affordability, or estimating costs associated with a program implemented by GHS, may serve as a “next step” in the research, rather than something that is fundamental to the current study. |  |
| **[Results]** A table comparing financial versus economic costs by the various stages (Annualized set-up and 1-year implementation costs) would be important for policy makers. Include both fixed and variable costs (i.e. capital, overheads, training, transport). | We have added a table comparing reported infrastructure and operational costs associated with the intervention. | 11 |
| **[Discussion]** The discussion should consider implication for the Ghanaian government. In the introduction the authors note the importance of this intervention for Ghana, but the opening statements in this section refer to impact of this programme for the donor only. | Per our response to the reviewer’s related suggestion in the Introduction section, the authors acknowledge the role that this analysis plays for Ghanaian policy- and decision-makers, as made clear by the active role Ghana Health Services is playing in scaling up this intervention. Language has been added to the manuscript to reflect implications of this analysis for the government. | 23 |
| **[Discussion]** How does the cost per DALYs averted in this programme compare to others economic evaluations of programmes aimed at reducing neo-natal mortality more broadly in this region? | Due to the heterogeneity across contexts, it is difficult to compare the intervention discussed in this manuscript to other interventions that also address neonatal mortality throughout sub-Saharan Africa. Comparing the cost effectiveness of an intervention for training traditional birth attendants, for example, to the systems-strengthening intervention presented here would not provide useful insights.  With that in mind, we have added additional language in the manuscript discussing an aligned training intervention and the costs associated solely with the training component. In comparison to a purely training-focused intervention, the systems strengthening approached discussed here proves to be significantly more cost effective.  Finally, as mentioned in response to the reviewer’s comments regarding the Introduction, we have added additional literature about the cost effectiveness of other neonatal mortality interventions and the gaps filled by this analysis. | 5-6 |
| **[Discussion]** It is unclear why the authors indicate this study “does not lend itself to policy decisions”. As this programme was part of the broader government MEBCI, surely it has implications for the Ghanaian MOH ? Given current global funding issues, some analysis should be conducted to discuss implications around affordability and sustainability. | Per our response to the reviewer’s related suggestion in the Introduction section, the authors acknowledge the role that this analysis plays for Ghanaian policy- and decision-makers, as made clear by the active role Ghana Health Services is playing in scaling up this intervention. Language has been added to the manuscript to reflect implications of this analysis for the government. | 23 |
| **[Discussion]** Some discussion on the generalizability of the findings and key biases should be discussed. | A discussion of generalizability of these findings has been added to the manuscript. | 23 |
| **[Discussion]** The authors highlight key limitations- some information on how this would have impacted (i.e. over-/under-estimated) the overall outcome (cost per DALYs averted) should be discussed | As discussed in the Methods section, using the steady-state assumption to estimate a counterfactual is likely to create an over-estimation of the number of neonatal deaths averted by the intervention. Additionally, we have added text to the Discussion to clarify potential over- and under-estimations of analysis under different assumptions. | 22 |
